# “I’m not your grandpa”: Experiences of advanced age fathers raising their teenage children in Belgium, an interpretative phenomenological analysis

**DOI:** 10.1371/journal.pone.0309448

**Published:** 2024-08-26

**Authors:** Steven R. Piek, Kato Verghote, Andrea Martani, Guido Pennings, Veerle Provoost

**Affiliations:** 1 Department of Philosophy and Moral Sciences, Bioethics Institute Ghent, Ghent University, Ghent, Belgium; 2 Institute for Biomedical Ethics, University of Basel, Basel, Switzerland; Ministry of Health, Sri Lanka, SRI LANKA

## Abstract

This study aims to gain more insight in the lived experience of men who became father at an advanced age (40 years or older). Advanced Parental Age (APA) is becoming an increasingly widespread phenomenon as the average age at which people have children has been increasing for decades now. However, the psychosocial dimension of APA-fatherhood in particular remains a highly understudied topic. This Interpretive Phenomenological Analysis presents findings from a qualitative interview study with seven men who fathered their (now teenage) children in their early 40s to early 50s. Interviews were semi-structured and focused on lived experiences of the participants and their normative stances regarding the topic of parenting at an advanced age. Three themes were identified: The fathers in our sample describe their APA as a result of life events rather than an intentional postponement. Second, they managed how they were perceived as APA-fathers by distancing themselves from ‘too old’ parents. However, these fathers did not perceive fatherhood at a younger age as better than their current APA. Three fathers, who also had an earlier fatherhood experience, provided a rich account of how they made sense of their fatherhood roles in both families. Third, the seven fathers encountered social stigma, leading to various coping strategies. These findings contribute to better understanding the psychosocial dimension of APA-fatherhood.

## Introduction

The number of men and women having children at advanced age has been increasing in Western countries over the last few decades. While late births are not a new phenomenon historically, first and second-order late births are [1,2]. Across seventeen Western countries, the average proportion of first-time fathers aged over 45 has risen from 2.48% in 1990 to 4.11% in 2014 [2]. Between 1972 and 2015, the average age at which men fathered a child in the United States increased from 27.4 to 30.9 years [3]. In European countries, this average is even higher, with 33.3 years in Finland and 34.7 years in Germany, for example [4,5]. The average age for women is slightly lower, but increases at a similar rate. This ‘postponement transition’ [6] has drawn much attention to the negative biomedical implications that having children at Advanced Parental Age (APA) potentially has for women and their offspring [7]. Additionally, there has been an increasing interest in exploring the impact of *paternal* age on fertility and the health of their offspring [8].

However, conducting research to better understand the many facets of the phenomenon of APA is challenging for many reasons. One of the major ones is the lack of consensus about the onset of APA, which contributes significantly to the difficulty of drawing conclusions from existing research. Defining APA for men is even more difficult than for women, since men’s reproductive capacities decline in a different way. Thresholds used in existing research investigating the consequences of APA for men vary widely. Depending on the focus and framing of a given study, these may range from 30, to 40 and even 55-years-of-age [8,9]. Additionally, one study may use clearly demarcated age-groups to study the impact of advanc*ed* paternal age, while another may work with advanc*ing* paternal age as a continuous parameter [10]. Existing empirical evidence shows that as men age, sperm quality tends to decrease, which is associated with lower pregnancy- and live birth rates [11], and leads to slightly increased risks of conditions in the offspring such as Autism Spectrum Disorder and schizophrenia [9,12]. However, these risks associated with male reproductive ageing remain low, particularly when compared to the baseline risks of genetic defects in offspring.

Even if it is difficult to determine from which exact age fathers can be defined as having an APA from a biomedical perspective, societal perceptions on life stages may nevertheless define some people as ‘too old’ to have children. For example, the European Social Survey conducted in 2018–2019 found that people believe the social age-limit for men to father children spans between in the late 40s and around 50-years-of-age [13]. Belgian respondents expressed the lowest threshold, holding that men above 46 years are too old to become fathers [14]. However, the influence of ‘chrononormative’ [15] ideas is declining because individualisation, secularisation and the loosening of normative constraints weaken the influence of such norms on family life. The evolution of this ‘Second-Demographic Transition’ varies greatly across societies and cohorts, so that social norms (e.g., regarding the ‘right’ age to parent) still play a role in guiding demographic behaviour [16].

The vast majority of empirical research on the psychosocial dimension of APA and family behaviour in general has focused on mothers. Meanwhile, the few studies that did focus on fathers and age, date back one to several decades (e.g., A 1994 study with data from 1988 [17], or a 2010 study with data from 1999 and 2000 [18]), included fathers of newborns and did not specifically look at APA (e.g., [19]), and were only from the US and the UK. In other words: Even though there are a few (dated) studies on fatherhood and age, those did not focus on the lived experience of fathers of *advanced* age. Hence, there is a significant gap in the available research. In this context of a lack of attention for the lived experiences of fathers, our study aims to contribute to the knowledge and understanding of APA by providing recent insights into the experiences of men who became parents at an APA. Based on the in-depth analysis of seven interviews with APA-fathers, we set out to investigate how they perceive and make sense of the circumstances that led to their parenthood timing. In addition, we explored how they coped with social judgement, social norms (i.e., social age-limits), and their personal norms, including what they consider to be the right time to have children, and for what reasons.

## Method

This study is part of the A-PAGE project, a Swiss-Belgian interdisciplinary research project on Family Building at Advanced Parental Age. The project aims to increase insight into the experiences, the moral reasoning and the decision-making processes of parents of advanced age, children born to such parents, and professionals. The project explores the moral, legal and social significance of age as a factor in these families and in family building in general.

For this paper, we adopted a qualitative approach and chose Interpretative Phenomenological Analysis (IPA) as our analytical method. IPA aims to gain an understanding of participants’ lived experiences and typically involves sample sizes ranging from five to fifteen [20]. As a result, it is possible to explore dimensions of their lives and meaning-making. The aim of this study was therefore not to make representative claims at the population level, but rather to broaden the debate by presenting the perspectives of those directly involved.

### Recruitment

As illustrated in the introduction, there is much debate on the exact age from which fathers can be considered of APA. Our choice of 40+ years was made to better align with empirical research on the psychosocial dimension of motherhood at an APA, where 35–40 are often used as threshold ages. Moreover, 40 is the threshold that is nowadays most frequently used in the literature investigating the phenomenon of APA-fatherhood [8]. Additionally, we differentiated between first-time APA-fathers (men who became fathers for the first time after turning 40) and ‘renewed’ APA-fathers (men who fathered children after 40 but also did so at least 10 years earlier in their life). The latter resembles ‘renewed-mothers’ as introduced by Jarvie et al., who came up with this term to challenge “the predominant framing of ‘older’ motherhood as problematic” [21, p. 104]. To be included in the study, our participants had to have fathered at least one child while being 40 years or older (regardless of whether they already had children). This child had to be between 12 and 18 years (i.e.: teenager) at the time of interview and live together with the parent(s). We focused on this age group because these fathers have accumulated more actual parenting experience than fathers of newborns or toddlers. We would exclude those not being significantly involved in raising their child (e.g.: divorced fathers living abroad) We collected our sample in Flanders, the Dutch-speaking part of Belgium.

The seven participants were recruited via letters distributed through high schools (n = 6) and the Student Guidance Centre of Flanders (n = 1) (The Student Guidance Centre provides high-school students with guidance and support throughout their education. The organization is connected to all schools in Flanders, but functions independently). Semi-structured interviews, lasting an average of one and a half hours, were conducted by the first author between January 21^st^, 2022 and February 17^th^, 2023. The interviewer had extensive experience with interviewing because of his background in journalism. Prior to the data collection, he received training that specifically focussed on interviewing for qualitative research. The Ethics Committee of the Faculty of Arts and Philosophy at Ghent University approved this study (Ref. 2021–33), and each participant provided written informed consent before the interview began. The interviews were conducted face-to-face and in a private setting, either at the participant’s home, workplace, or the Bioethics Institute Ghent. We recorded and transcribed the conversations verbatim while pseudonymizing all names and locations mentioned.

### Interview procedure

The interviews were structured into two parts. In the first part, the interviewer explored the participant’s experiences with becoming a father later in life, fatherhood, interactions with and reactions from their social environment, and his status as an APA-father. The second part of the interview focused on the participant’s moral reasoning about age and parenting in a more abstract, general sense. To encourage this, we employed two ‘elicitation techniques’ similar to those used in previous research on lay people’s reproductive decision-making [22,23]. An elicitation technique is a means, usually in the form of a task, to encourage an interviewee to think more abstractly about topics related to the study. We incorporated visual elements in these tasks on purpose, as they typically “enhance the ability to ‘draw forth’ valuable knowledge from the participant” [24, p. 7].

**Fig 1:**
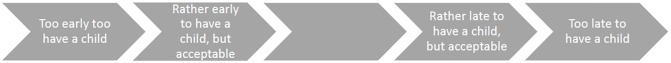
Normative timeline. During the second half of the interview, participants were handed this normative timeline printed on a piece of paper. They were then asked to write down specific ages or -ranges (referred to as ‘age-markers’ in the text).

The first elicitation technique involved a printed timeline divided into five stages representing sections on a normative continuum, ranging from ‘too early to have a child’ to ‘too late to have a child’ (see Fig 1). Participants were asked to write down specific ages or age ranges (referred to as ‘age-markers’ below) on this normative scale. These ages could have been any age (range) that for them would represent a ‘limit’ at which they thought the moral acceptability of conceiving a child would change. They were encouraged to ‘think out loud’ while completing this ‘timeline-task’. The instructions intentionally did not specify a gender, allowing participants to make this distinction themselves if they deemed it relevant. If the participant did not make a distinction between men and women, the interviewer asked them to consider the possibility of such a distinction.

For the second elicitation technique, the interviewer handed printed cards with various normative statements about age and family building or parenting. Participants were then asked to categorise each statement by laying the cards on a pre-printed sheet of paper with two columns (‘agreed’ or ‘disagreed’, and the possibility to also place the card in-between them) and were again encouraged to think out loud and explain their views regarding each statement.

### Analysis

IPA is a research method that aims at gaining an understanding of the participants’ experience of a specific aspect of their lives, while at the same time recognizing the mutual construction of such accounts by both participants and researchers. As such, IPA draws on both the traditions of phenomenology and hermeneutics [25,26]: The phenomenological element lies in its “emphasis on the experiential claims and concerns of the persons taking part in the study” [25, p. 104]. The hermeneutic element, on the other hand, involves the subsequent act of interpreting the data in a broader socio-cultural and theoretical context. This allows researchers to approach the data in a more interpretive way, thinking “about ‘what it means’ for participants to have made these claims and to have expressed these feelings and concerns in this particular situation” [25, p.104].

For this particular study, the first author began by reading through the transcripts and keeping track of any relevant associations and initial impressions, also known as ‘memo-writing’. In a subsequent stage, the software NVivo (v.1.6.1) was used to organize the analysis process. After establishing a tentative thematic structure of the data, multiple ‘auditing rounds’ were conducted [27]. During these rounds, the fourth and fifth, and later the second and third authors, provided their perspectives and ideas on the selection and thematization of the interview data. This sparked discussions that helped to improve the structuring of themes and prevent tunnel vision on certain interpretations. The auditors’ diverse academic backgrounds, including philosophy, gerontology, journalism and law, also made a broader interpretation of the data possible. Following each auditing round, the first author reviewed the data and reconsidered the arrangement of themes and codes. Eventually, the first author contextualised the participants’ experiential accounts in a broader sociocultural and theoretical context (cf. Discussion).

## Results

We conducted interviews with seven APA-fathers corresponding to the inclusion criteria as described above (See Table 1 for an overview of our sample). The fathers had children who were teenagers (between 12 and 18 years old) at the time of the interview (In Belgium, children attend high school from the age of 12 onwards). Of the seven participants, three were renewed fathers, while the remaining four were first-time APA-fathers. All participants were heterosexual and of Caucasian ethnicity. One father had his child through IVF and the others through natural conception. Four fathers had no religious affiliation, one identified as agnostic, one as Christian and one said he had faith but no specific religion. Two completed secondary education and five obtained a Bachelor’s or Master’s degree.

**Table 1:** Participant characteristics.

Pseudonym	ATOI	CFAA	CFYA
Gert	60	2, late teenage years	n.a.
Chuck	59	1, mid-teenage years^a^	n.a.
Michael	60	2, mid- and late teenage years	n.a.
Sam	59	1, mid-teenage years	n.a.
John	67	1, mid-teenage years	1, during mid-20s
Simon	58	2, early- and mid-teenage years	2, during early- and mid-20s
Jasper	58	1, early teenage years	2, during late 20s and early 30s

ATOI = Age at Time Of Interview (Some ages were altered with -1 or +1 year to reduce the risk of identification), CFAA = Children Fathered at Advanced Age (Early teenage years means 12–13, mid-teenage years means 14–16, and late teenage years means 17–18), CFYA = Children Fathered at a Young Age.

^a^ = Chuck had been raising one stepchild since toddler-age.

**Fig 2:**
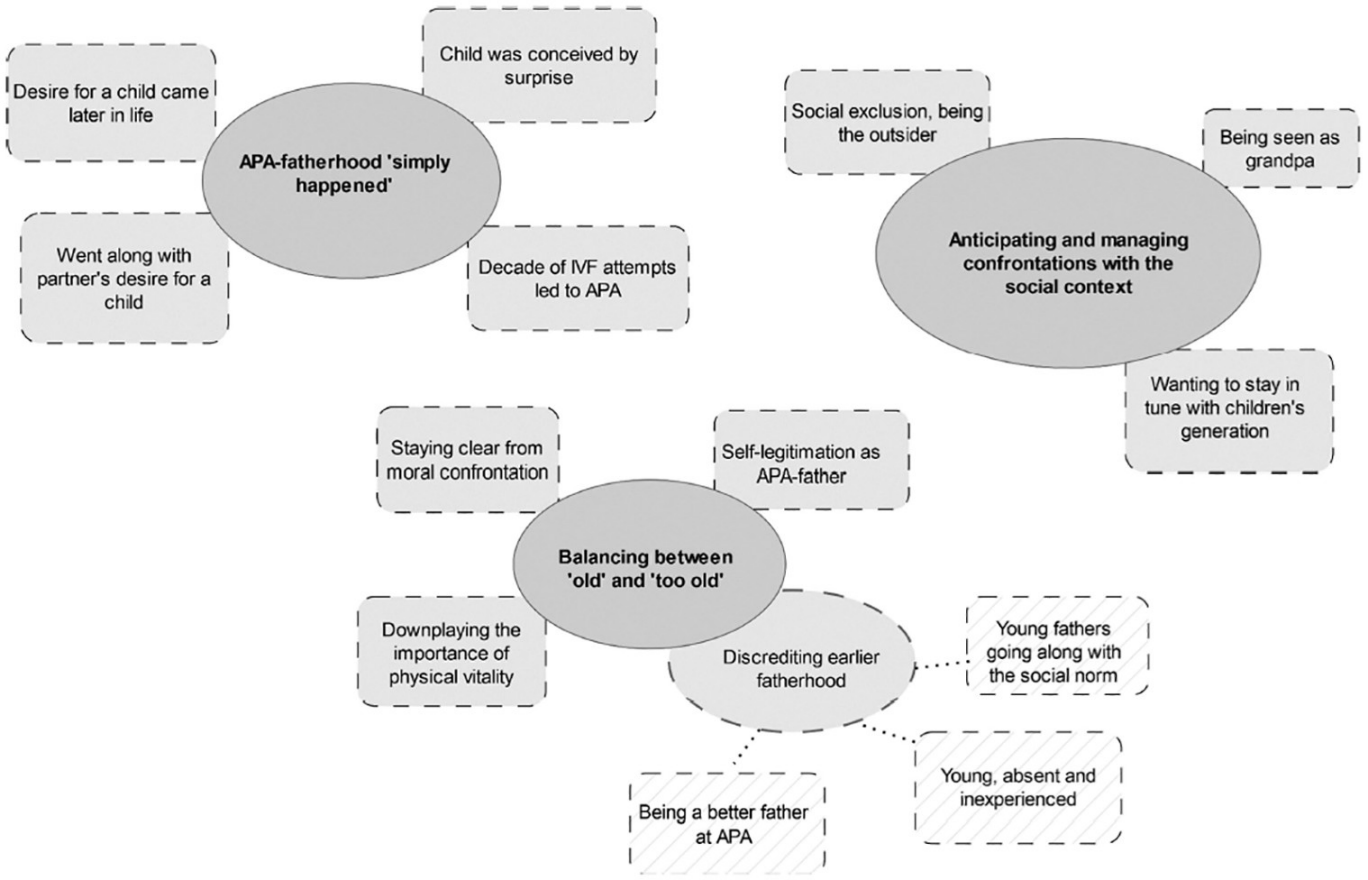
Thematic map; legend/caption: Visual representation of the themes and their related codes.

In what follows, we present three themes and one sub-theme that explore how our participants experienced their APA and how they think about the topic in general (see Fig 2). In the first theme, it is shown how the fathers in our sample describe their APA as a result of life events rather than an intentional postponement. The second theme explores how the participants dealt with others’ perception of them as APA-fathers by taking distance from the label ‘*too* old’ while at the same time underlining the benefits associated with APA. In the second theme’s sub-theme, the three renewed fathers compare their young fatherhood with their current experience and express their preference for the latter. The third and last theme shows how the fathers in our sample coped with the social stigma they anticipated and encountered, leading to various coping strategies.

### 1—Fatherhood ‘simply happened’

For the fathers in our study, deliberate timing and intentionally postponing parenthood as such, barely played a role in their decision to become father after the age of 40. Instead, they mentioned specific life events and unforeseen circumstances when they explained what led to their advanced age fatherhood. For example, Chuck had been a stepfather in the past during several brief relationships, but he had never considered fathering a child himself. However, after meeting his current partner who was told that she could not have children, Chuck experienced the conception of his daughter as a surprise. His fatherhood thus ‘simply happened’, since the fact of becoming a father at advanced age was not linked to a decision or intention to have children late. Moreover, the interview setting may have given the participants a feeling of having to retrospectively *justify* their APA. For instance, when Chuck was asked to describe the relationship between him and his child, he spontaneously said:

“You can always say: ‘Why didn’t I have children earlier?’. It just never happened, the occasion to have children was just never there”

–Chuck

We saw a similar pattern in the accounts of all participants: their APA ‘arose’ from their life course almost as an unintended ‘side-effect’. Michael became father in his 40s because he and his wife had first been trying to conceive via IVF for almost a decade, after which they unexpectedly conceived twice through natural reproduction. Sam and his wife simply did not desire to have a child for a long time and when they eventually did, they needed IVF treatment. The APA of Jasper and John resulted from the fact that they met their current wife at a later stage in their lives. Four fathers indicated that they saw their own timing as sub-optimal, but they did not regard it as a conscious choice of postponement.

#### Going along with the partner’s desire

Simon and Gert stood out in terms of how their APA came about, as neither of them really wanted a child in the first place. Instead, they attributed this desire to their partners. Gert expressed that he has “always been amazed about men who have a very strong emotional longing for children”, and Simon was quick to point out that in neither of his two relationships where he fathered children did the desire for a child come from him. He even made a joke about it:

“I sometimes say: ‘If it were up to men, we might have gone extinct a long time ago’.”

–Simon

When confronted with his wife’s desire for a second child while he was already at APA, Simon pushed the question forward for a few additional years. He associated the possibility of becoming a father again with fears related to his “somewhat traumatic” fatherhood experience back in his 20s, stating that he “was really afraid of that”. According to Simon, his change of heart was caused by his wife, who at some point seriously urged him to reconsider, stressing her strong desire to have another child. When asked what ultimately tipped the scales, he said:

“[M]y love for her, yes, I think that, well, it’s very strong. With her I am a very happy person and I really think that was the decisive factor. […] Also reconsidering it like: ‘What does it mean for her?’ and, um, and, it’s easy for me to say ‘No’, but, well…”

–Simon

Like Simon, Gert mostly just went along with his wife’s wish to have children. Despite leading busy and tumultuous lives in their 30s, Gert and his wife had initially planned to remain childless, believing that having children was more suitable for people in their 20s. However, Gert said that when her youngest nephew was born, her desire to have children ‘kicked in’, and her perspective changed:

“It boiled down to me finding it very selfish at a time when Emma’s biological alarm clock started ringing, um, to say: ‘No! I don’t want to’.”

–Gert

### 2—Balancing between ‘old’ and ‘too old’

Each father in our sample expressed their awareness of being older than the usual age men have children at. We noticed that they reconciled with such awareness in either of two ways: 1) they stressed that everything was alright in their fatherhood journey and, by doing so, implied that their parenthood was ordinary even if their age was not; or 2) they openly acknowledged that their APA somewhat put them in the position of an outsider, made their parenthood ‘special’ or ‘unordinary’, but subsequently reframed this ‘un-ordinariness’ in a positive way. All three renewed fathers at some point referred to themselves as “belonging to the grandpa category” (Jasper) or (soon) being ‘an elderly father’ (Simon, John). In contrast, the four first-time APA-fathers tended to emphasize that they were not really different from younger fathers. Overall, we observed a peculiar tension: The fathers downplayed the importance of age if it would imply something negative about their fatherhood, whereas they *did* attribute meaning to (advanced) age when they could associate it with certain upsides and benefits.

#### Staying away from the stereotype of an old father

The first-time older fathers (Gert, Michael, Sam & Chuck), were particularly keen to avoid being perceived as ‘too old to be a father’. At the start of the interview with Michael, the interviewer perceived that this participant anticipated scrutiny and was inclined to respond optimistically. For instance, when asked a question without any prompt for a positive or negative response, Michael immediately replied that everything was ‘going perfectly well’ (“That works out perfectly” and “Yes, that goes perfectly”).

Michael, Sam, and Gert each emphasized that they did not consider themselves to be ‘out of touch’ with their children, distancing themselves from the image (or stereotype) of a disconnected older father. The following quotes demonstrate how they asserted their capacity of being actively involved in their children’s lives.

“I, well, I have a bit of a sense of what’s going on in the world of these youngsters. (…) [W]hen I go along, uh, to the football, same story: I can level with those guys, I can tell them something silly now and then,… There are a lot of people who are younger than me, who go to the football, all so *serious*… [with a bored tone]”

- Michael

“I’m now, um, 60, so I think I still have the mental fitness to engage in discussions with both my daughter and son, which is a bit more challenging with the son. With the daughter, it’s getting a bit easier, but well. There’s a kind of equality to be able to debate.”

–Gert

Gert’s phrasing may suggest that he believed his advanced age could impact his mental fitness, thus *potentially* making him unable to meet the standard of a ‘good parent’. Interestingly, except for Chuck, who at some point suggested that his health problems might be related to his age, none of the fathers addressed the topic of physical ageing on their own initiative. When, during the elicitation technique, they were presented with the statement ‘*If a father cannot play football with his child in the park*, *then he is too old’*, all participants disagreed. They either disregarded the importance of physical activity for being a good father altogether, or rejected the association between physical fitness and ‘being too old’. None of the participants mentioned making a conscious effort to counteract the effects of aging on their physical ability.

#### Avoiding a moral confrontation about their fatherhood timing

As mentioned before, especially the first-time APA-fathers stressed that they did not deviate from the norm or fell short in what they offered their children compared to younger fathers. However, the way they talked suggested that they felt confronted about their age, thus feeling a need to justify themselves. Chuck, for instance, stipulated that the appropriate timing of parenthood is not determined by age. Yet, he did mention “problems” related to his APA, which at the same time helped him to bond with another APA-father:

“Also, it was easy to talk about it [with him], like it’s a bit the same… Similar problems and such.” […] “Yes, also, um, no grandparents anymore and yes, there was immediately some friendship and it has been easy, um, that suddenly, yeah, you see people who are in the same situation.”

–Chuck

Chuck felt a sense of familiarity and acceptance through this connection, suggesting that his APA may have caused him to feel somewhat alienated from other parents.

Finally, it is worth noting that during the timeline-task, each participant who formulated an age-marker for ‘too late to have a child’, picked an age higher than their own at the time they fathered their lastborn (see Table 2). Jasper, who had his last child at the age of 44, made it quite clear that he wanted to avoid a moral confrontation about his own timing:

Jasper: “‘Rather late’, I find it rather late at… 45. And ‘too late’, yeah, I *must* put 55 because otherwise, it wouldn’t make sense.”

Int: “Why wouldn’t it make sense otherwise?”

Jasper: “Yeah, because then I’m past it [laughs]!”

**Table 2:** Overview of the 7 fathers who we interviewed and their age at the time of the interview, juxtaposed to from what age onwards they thought it would be ‘rather late’ and ‘too late’ to father a child (typically expressed during the timeline-task).

Participant’s age			Age Markers for Late Parenthood Mentioned	
Pseudonym	ATOI	ATOI	‘Rather late, but acceptable’	‘Too late to have a child’
Gert	60	43	≥35	≥50
Chuck	59	45	≥60^a^^b^	≥70^a^^b^
Michael	60	47	≥40	≥50
Sam	59	44	refused^b^	refused^b^
John	67	52	≥50	≥60
Simon	58	48	≥50^a^^b^	≥55^a^^b^
Jasper	58	44	≥45	≥55

ATOI = At time of interview, ABLC = At birth of last child,

^a^ = Placed this age marker with much reluctance,

^b^ = Repeatedly stated not wanting to judge about others because every decision is context-dependent and every situation is unique.

All participants placed the age marker for ‘too late to have a child’ beyond their own age, which, on the contrary, fell in the category of ‘late, but acceptable’. In so doing, they accepted the label of ‘old fathers’ (and often stressed the advantages of being older than average), but also insisted that they distanced themselves from the idea of being ‘*too* old to have children’. Some even disconnected the label ‘too old’ from the factor ‘age’ altogether. A good example of this is the moment when Sam contemplated the timeline-task, during which he refused to formulate age-limits:

“*I* know that [determining what is ‘too old’] is much more complex than [mentioning an age-limit]. Because it depends on much different factors than applying statistical data. It also makes me nervous, if people would do that, like ‘yes, now you are too old to have a child’. Then I will say ‘yes, and that fort- that thirty-something is not too old, or what? But he divorced twice and hits his children!’”

–Sam

### 2.1—Discrediting earlier fatherhood

When the three renewed fathers (Jasper, Simon & John) compared their younger fatherhood experiences with their APA-fatherhood, they all described the latter as more desirable. Jasper, who became a father again at the age of 44, expressed great enthusiasm about now being much more involved with his youngest daughter. Similarly, Simon and John’s APA-fatherhood experiences were in stark contrast to their fatherhood experiences in their 20s.

“[My older children] did not have that fortune [of a balanced mother]. That caused quite some upheaval… I always feel like, well: ‘[My older children] really had to swallow a lot, and [my younger children], so to speak, are getting it, yes, effortlessly’, how smoothly it all goes. […] So with the younger children, as I am also older, with a lot of life experience, having learned a bit: ‘Okay, this is important, this is not important, these are boundaries that need to be maintained’. Those [younger] children also have a bit more structure than [my older children], because back then, I was very young, I was super young, what did I know?”

–Simon

The three renewed fathers each indicated that when they had children in their 20s, their social context normalized or even encouraged the timing of their fatherhood:

“Back then, you were young, it was expected of you, it’s also normal, well, you kind of go along with that flow.”

–Jasper

“Married, um, a year or two later, a baby comes along, uh… Yeah, that was just how it was.”

–John

Despite conforming to social expectations about family planning at the time, these men described their experiences as fathers in their 20s as far from ideal. John recalled how his first marriage and the birth of his first child happened too quickly, which he referred to as a “mistake” and something that “should never have happened”. He stated that his relationship was far from good and that, at 23, he wasn’t ready for fatherhood. Simon remembered how his difficult childhood and his first wife’s psychological problems affected the well-being of their first children. When Jasper was asked how he had experienced his first-time fatherhood, he resolutely answered: “I didn’t [experience it at all]”. He also had no recollection of his eldest children’s first steps, their first words, nor their daily lives apart from playing with them:

“[S]chool, sports camps, um, or sports or hobbies in general, I [knew] nothing about that. I [knew] *nothing*, absolutely nothing about that.”

–Jasper

These three renewed-father experienced their APA-fatherhood much more positively than their earlier fatherhood. They attributed this to having more life experience, a better relationship, a more stable financial situation, and more time to spend with their children.

While the four first-time older fathers did not have the actual experience of being a young father themselves, they nonetheless associated similar benefits with APA.

For instance, Gert pointed out that having children later in life has allowed him to expose them to cultural experiences such as opera, theatre, and classical music. He also credited the APA of him and his wife for the resilience of their relationship, which endured the challenges they encountered while raising their children:

“[I] think that for us, the being older has, in a way, protected us [so] that we could somehow channel the difficulties that [parenthood] has brought along. And, actually, yes, having been able to endure it.”

–Gert

Sam and Chuck mentioned having more peace, wisdom and life experience. All participants expressed appreciation for being in a more financially stable position and having more time than younger parents would have. Chuck, for instance, said:

“I’m no longer in the rat race, uh, I don’t have to look for another job, I’m not going to do that anymore. […] You can also look at everything more calmly. The house is paid off, everything is peaceful, no problems with paying the bills.”

–Chuck

### 3—Anticipating and managing confrontations with the social context

Except for Gert, all participants were at some point in their day-to-day lives confronted with being different from other fathers because of their APA. Sam and Michael were mistaken for their child’s grandfather (quote presented below), Jasper noticed that people were sometimes unsure of his relation to his youngest child, and Chuck and John referred to confronting moments with the younger parents of their children’s schoolmates. For Simon, this confrontation occurred during the interview itself. While engaging with the timeline-task, with a degree of concern he suddenly envisioned himself in the near future as “an elderly father”.

We observed that some brushed off the experience of being perceived as an older father, while John and Sam expressed concern about how their children perceived them: John tried to avoid being seen as an old man to prevent his son from having a negative image of him, while Sam did it for his self-image.

#### Troubled by social judgement

During the interview, Sam appeared to be concerned about how others perceived him. This became particularly clear when he recalled an anecdote about his son calling him ‘granddad’. That comment had struck a chord with Sam, and he seemed to become agitated again while recounting this anecdote:

“[My son] had to talk about the family at school and then had to mention the age of his father, to then hear: ‘an old dad’. That did happen. Then he came home, [a]nd my son, he doesn’t do it anymore now, but every once in a while he said, he once said to me, ‘pepe’. That’s grandpa in our dialect. He said something like that, probably because others said, ‘yeah, your dad is old, and he’s like a grandpa,’ and he said that. And then I said, ‘you must never say that again. I’m not your grandpa, I’m your dad. Yes, I am an older dad, I am older than other dads, but I am not your grandpa. That people perceive me [like that], that’s a fact, I can’t do anything about that, and that’s just what it is, I can’t do anything about that’.”

–Sam

It is noteworthy that Sam speculated that his son’s peers must have suggested that his father is a granddad instead of the thought coming from his son. Also, he was quick to change “every once in a while he [my son] said” to “he [my son] once said”, as if he attempted to ‘soften’ the event. Additionally, although he admitted being older than the average father, Sam also stood out in his concern about how he was perceived by members of his son’s generation:

“I used to teach. I once ran into some former students during a [techno] concert and they would look at me like ‘what are you doing here?!’. ‘Well, I love this music and I come to a concert’ and then they were stunned. […] What do you think, that I would only listen to classical music or so?”

–Sam

Chuck also faced some social stigma due to his age, but in a different manner. He recalled being socially excluded and gossiped about by his peers at the local hobby club. They criticised him for becoming an unmarried father at the age of 45 and not baptising his child. At that point, Chuck chose to distance himself from those who looked down on him. Unlike Sam, he did not seem to be overly concerned about how he was perceived by others and shrugged when he spoke about this experience. While Chuck found it uncomfortable to see some of his former classmates waiting at the school gate with their grandchildren when he was there to pick up his toddler daughter, he brushed it off, saying “I looked at it once and moved on”. Similarly, John and his wife also experienced social exclusion. They were not invited to gatherings organized by the parents of his son’s peers. John mostly saw this as unfortunate for his son and attributed it to him and his wife “not really belonging” and being “from a different generation” than the other parents. He actively tried to compensate for the effects of ageing and expressed his desire to keep up with things for his son, as this is part of what a parent should offer to a child according to him. He gave an example by sharing an anecdote about his son’s excitement over the new family car’s features. Although John did not feel any enthusiastic, he felt a sense of responsibility to feign it for his son. Yet at the same time, John did not seem to be perceived as an ‘old man’ by his son:

“[Y]esterday, we were watching television […] Well, it boiled down to this, like *‘what do they still want, those old folks’*—the person might have been around 60 years old–*‘doing on TV?!*’ […] But [my son] overlooks that I’m actually even older, but he sees it differently, eh. I mean… But sometimes you say, you confront him, like ‘Charley, I am actually older than those people, you know.’ Yes, um, y- see, that’s the image of, uh… […] I am the burden of society [laughs].”

–John

Thoughts about his ageing process kept John awake at night sometimes and Simon and Chuck expressed concern about their advanced age as well. Chuck, for instance, admitted that his health problems were probably due to his age.

#### Concerns about the future

In the case of Sam and John (presented above), the societal expectations about fatherhood and age shaped the dynamic between father and son, while for Simon and Jasper (in the following) these societal expectations shaped their self-image. During the timeline-task, for instance, Simon expressed worry when he suddenly started contemplating his age by the time he would be picking up his youngest son from parties or other social events:

“I was almost 50 [when my youngest son was born], which means that if [he] is 20, I’ll be 70. You really think about these things. […] [W]hen he starts going out in six or seven years, and I have to pick him up or do something like that, well, you’re dealing with an elderly father who is in his late 60s you see, and, uh, that still has to match a bit. If the age difference is too large, it seems difficult to tolerate each other’s worlds, you know. Because you do live together. They’re not your grandchildren, after all.”

–Simon

Simon was quick to distance himself from this notion of ‘an elderly father’ by speaking in the second person. Although brief, these moments are interesting because at many other points in the conversation, both Chuck and Simon repeatedly stated that age is basically irrelevant to good parenting.

#### Disregarding social judgement

Not all fathers seemed to be affected by how others would perceive them. Michael said that he could laugh about being mistaken for his sons’ grandfather, and Chuck and Jasper shrugged off the judgments of others, attributing them to conservative societal standards. When asked whether he experienced being “in the grandpa category” as something negative, Jasper nonchalantly said:

“[F]or me, grandpa, no. At the moment when I decided to, uh, to say: ‘okay, we want to have a child’, I had already reconciled with that.”

–Jasper

For Jasper, being active and involved with his daughter was more important than being seen as a ‘grandpa’, despite the societal prejudice. He even made a generalization about this:

“[Those other, younger, fathers], they will miss a bit of what I have missed too, and they will only know that later. And that’s unfortunate.”

–Jasper

## Discussion

For the men in this study, it was never their intention to become APA-fathers, and four of them even explicitly stated that, in their view, the ‘ideal age’ for fathering a child was earlier than the one they had when they had a child themselves. They did not willingly postpone fatherhood. Rather, life events and unforeseen circumstances led to their APA. At first sight, this seems to be at odds with observations made by Henwood et al. [18], who interviewed 30 first-time fathers about their decisions to ‘delay’ having children. These men felt that by reproducing somewhat later, they would be better able to provide and be involved with all aspects of their children’s lives. Furthermore, they observed that the fathers in their study wanted to focus on their own development and career for a while, before putting someone else first. However, the men in the study by Henwood et al. [18] eventually had their children in their late 20s and early 30s and thus not at APA. And while their participants’ postponement was intentional, they did so with only for a few years and therewith became fathers at a still relatively young age. This might mean that ‘conscious postponement’ plays a role when it comes to deciding not to have children at a very young age and thus pushing it forward a few years, whereas it may be less relevant in cases of APA-fatherhood.

Given the scarcity of studies on APA-fathers, it is interesting to compare our results with those of studies conducted solely on APA-mothers. Observations made by Cooke et al. [28], based on their interviews with eighteen mothers of 35 and over who just became or were about to become mothers, show striking similarities with our findings pertaining the notion of ‘postponement’. Contrary to the idea that “women *choose* to delay having a baby due to education or career”, the women in their sample suggested that, instead, external factors “determine when they *can* choose to have a baby” [28, p. 37, emphasis added].

We observed that many fathers felt that they were perceived as ‘different’ from younger fathers and were aware that they did not meet the typical social expectations regarding the timing of their parenthood. Surveys conducted in European countries indicate that people generally consider the ideal age for men to father their first child to be in their mid-20s [29,30]. This age is increasing towards one’s late-20s [16]. However, the actual influence of such chrononormative attitudes held by members of society on the reproductive behaviour of others may be declining as a result of the Second-Demographic Transition [16] (cf. Introduction). In addition, all fathers in our sample appreciated their APA for, among other things, having more financial stability and life experience. This has also been observed in other qualitative studies on APA-fathers and -mothers [31,32]. Our participants also reported having more time and attention for their children, which is in line with a quantitative study by Heath based on 1988 US data [16]. Heath found that men who became fathers between the ages of 36 and 63 spent more time with their children and were more nurturing towards them than their younger counterparts. Our participants’ views about having better romantic relationships however, are at odds with observations made by Jadva et al. [10]. In their interview study, which included fathers and mothers between 30- and 60-years-of-age, they observed variations in relationship quality linked to parental age, indicating that mothers and fathers aged over 45 often experienced a lower quality relationship.

Although some of the fathers in our study said that it was important for them to remain in touch with their children and a few expressed some degree of concern about their future as an older father, none of them underlined the need of making an effort to stay physically in shape. They considered the importance of physical vitality only when probed by the interviewer. Subsequently, it became clear that they did not ascribe significant normative weight to it and that it played a subordinate role for them in their own experience of fatherhood. This shows a remarkable difference with a study carried out by Shirani, who interviewed 53 fathers between the ages of 15 and 54 and explored their concerns and experiences of ageing [19]. Interestingly, anxiety about their ageing process was expressed by fathers across all age-categories, particularly with regards to an (anticipated) decline in their ability to be physically active with their child(ren) through sport. Some of the men in Shirani’s study even made a conscious effort to maintain their vitality [19]. However, the main difference between our study and the one of Shirani, is that the latter describes an intention of fathers just before and after the birth of their child, while our study describes an actual experience. Possibly, this earlier intention makes room for other priorities as time goes by and fathers and their children age.

As mentioned in the introduction, the mean social age-limit for aspiring fathers is in one’s late 40s, according to the European Social Survey of 2018–2019 [13]. Belgians were the strictest, in that they selected 46 as the social age-limit for men to have children [13,14]. However, the men in our sample, also Belgian, chose upper age-limits between 50 and 70 (see Table 2). This discrepancy may be due to our sample selection (i.e., older fathers, vs the general population in the surveys cited above), or to our framing of the question (which allowed participants to differentiate between ‘late but acceptable’ and ‘too late to have children’). Another explanation could be that our participants selected higher ages to keep doubts about the legitimacy of their own APA-fatherhood at bay, while at the same time using various discursive strategies (often not applied in an intentional way) to steer clear from the judgement ‘too old’. These observations are consistent with Ylänne & Nikander’s research, for which they interviewed 15 couples and individuals who had their first child between the ages of 35 and 57 [15]. In their discourse analysis, the authors examined how social norms regarding reproductive timing were met by APA-parents. Similar to the fathers in our study, Ylänne & Nikander’s interviewees positioned themselves higher in the moral hierarchy of ‘good parents’ than younger parents or their younger selves based on their acclaimed life experience [15]. Like the men in our sample, when faced with differences between the concept of an ‘ideal parent’ and their own age, they either used humour, dismissed the differences as irrelevant or focused on the advantages associated with APA.

### Limitations and strengths

First of all, since all participants were of Caucasian ethnicity, heterosexual and relatively affluent, our sample lacks demographic variety. As such, we did not have the opportunity to learn from the experiences of minorities and those in a more disadvantaged economic position. Self-selection bias forms another possible limitation, as APA-fathers with predominantly negative parenting experiences may have refrained from participating, thus potentially leaving important aspects of fatherhood at APA remain unnoticed. Finally, despite the interviewer’s efforts to maintain a broad focus on the topic of APA throughout each interview, it is possible that the approach of the study, which is centred on age, may have influenced the participants’ thought processes and responses.

The main strengths of this study are its focus on an under-studied group in the APA literature, as well as the richness of its data in general. The use of elicitation techniques was particularly valuable, since inviting the participants to ‘think out loud’ provided insights in the participants’ thought processes that would otherwise have remained obscured. Another strength of this study lies in the fact that the interviewees spoke on the basis of about fifteen years of actual parenting experience at APA, while most other interview studies about APA focus on parents of newborns who can thus only speculate about their future as APA-parents.

## Conclusion

This study’s findings offer insights into the lived experiences of APA-fathers who are raising their teenage children. In addition to exploring what led to their unintentionally postponed fatherhood, we have shown how such APA-fathers may attempt to positively influence how outsiders perceive them and how they anticipate and cope with stigmatising attitudes and reactions from their social context.

This study adds to the limited body of literature about the psychosocial dimension of APA-fatherhood, which is however an increasingly widespread phenomenon. We found that none of them had deliberately ‘postponed’ fatherhood. Instead, their unique life trajectories led to their unconventional timing of parenthood. We also noted that some struggled with (their fear of) being perceived as ‘granddad’, expressed concern about growing older in relation to their child(ren), or felt the need to assert themselves as competent fathers during the interviews. At the same time, however, these fathers mentioned few disadvantages associated with APA. For most participants, age had never really been an issue. Moreover, the three fathers who also had children in their 20s reported that they had a much better fatherhood experience and more time available for their children now at APA than as young fathers. Those who did not already have children when they were younger, reported similar benefits and expressed a preference for APA. We also observed how the fathers in our sample perceived and dealt with (negative) perceptions of others in their social context and how they hardly perceived their APA as problematic. Finally, our results indicate that the debate on the ethics of family planning should step away from the notion that people’s reproductive timing typically results from a deliberate and intentional decision making process, especially in case of parenthood at an advanced age. This study underscores the importance of focusing on men and their lived experience of fatherhood specifically. We thus believe that there is a pressing need for more research in this regard, carried out in a plurality of settings and with more diverse sample (e.g. in terms of ethnicity, income, education…). Additionally, further research could explore how aspiring APA-fathers see their role and responsibility in reproductive timing, since such discussions mostly focus on the female ‘biological clock’. Another interesting research avenue would be to investigate the reproductive decision-making process of APA-couples instead of studying men and women separately.
